# Students perception towards feedback in clinical sciences in an outcome-based integrated curriculum

**DOI:** 10.12669/pjms.343.15021

**Published:** 2018

**Authors:** Tahir Ansari, Ambreen Usmani

**Affiliations:** 1 Dr. Tahir Ansari, MCPS, FCPS, MRCPI. Department of Clinical Sciences, College of Medicine, Majmaah University, 11952 Almajmaah, Kingdom Saudi Arabia; 2 Prof. Ambreen Usmani, MPhil, MCPS-HPE. Department of Anatomy and Medical Education, Baharia University Medical and Dental College, Karachi, Pakistan

**Keywords:** Feedback, Constructive, Strategy, Outcome-based curriculum

## Abstract

**Background & Objective:**

Feedback has been identified as one of the key strategies for learning in the outcome-based curriculum. Students are more interested in their grades paying little attention to the feedback, may not understand the importance of feedback and its effect on their performance because of their perception, and beliefs. Non-constructive feedback will not result in the improvement of the students’ performance. This study aims to explore; student’s perception of useful feedback; the purpose of feedback and believes about written feedback.

**Methods:**

This analytical cross-sectional study was conducted from November 2017 to January 2018 at Majmaah University. Students studying in clinical phase were recruited. Data were collected from 121 students by self-structured questionnaire using complete enumeration sampling method.

**Results:**

Majority of the students (45.5%) disagreed that the feedback should always contain marks; (49.6%) commented that the tutor did not provide enough constructive feedback. While we ask the purpose of feedback (62.8%), agree with two-way nature of feedback, and it is helpful to find there expected performance. Almost two third (67.8%) of the students believe that limited feedback is the reason for frustration and they did not receive comments for improvement.

**Conclusions:**

Students are aware of the purpose of feedback. Senior students give more value to feedback and in the opinion that feedback provides useful suggestions for future improvement and limited feedback is the reason for frustration. The results highlight the need for more structured feedback mechanism, and there is a need for faculty engagement in training to fill the existing gapes to create an effective educational alliance.

## INTRODUCTION

Feedback is a key strategy for learning, teaching and improving outcome-based curriculum. Research on students’ perception of this highly effective strategy has been reported.^1^ Effective feedback practices provide a bridge between assessment and self-motivated study. This is a cost-effective approach to enhance student’s attitude towards learning^2^ and embracing the concepts of outcome-based education.^3^ Feedback is a two-way process, educator and student reach an agreement to achieve the educational outcome against a benchmark. The student’s perception, understanding the purpose and believes about feedback is the most important determinant for achieving that outcome.^1^,^4^ In the absence of effective feedback, a good practice is not reinforced, poor performance is not corrected, and the path to improvement is not identified.^5^ This is a substantial motivating factor in acquiring and developing clinical skills, communication skills and professional bedside manners.^6^

The lack of adequate amounts of effective feedback in the clinical setting has been identified as a significant ongoing problem in medical education.^7^ similarly; feedback given in an unprofessional and ineffective manner can also result in demotivation. Clinicians are familiar with the concept and principles of giving feedback mostly, but often it remains underused, which may be attributed to several factors, e.g., learning culture, relationships with peers and students and emotional response to feedback.^8^ however, it has been reported that students felt reassured and more comfortable and perform better than students who do not receive feedback. Such students have been exposed to this strategy from an early age and look forward to feedback so that they may improve their learning and psychomotor skills.^9^ which exist in medical colleges, students are more interested in their final assessment and pay little attention to feedback with a focus on marks or grade to avoid failure rather than excel.^10^ Feedback seeking behavior in the clinical setting improve learning, particularly if educator highlight the area for improvement. Educators need to ensure that students understand the feedback message.^8^,^10^ Students may not efficiently use feedback due to the misconception or limited understanding for how to utilize these comments for improvement, and there is need for a common understanding by both faculty and students for the future implication of feedback.^11^

This research will help in answering the existing gap between the actual and desired understanding of feedback during clinical years, which is required to create an educational alliance between students and faculty for better learning. Therefore, the objective of this study was to explore; student’s perception of useful feedback; student’s perception on purpose of feedback, and to find the students believes about written feedback.

## METHODS

The analytical cross-sectional study was conducted from November 2017 to January 2018 in College of Medicine, Majmaah University, KSA. Ethical approval was obtained by the ethical review board at Majmaah University (MUREC- Nov l4/COM-2017/23). All undergraduate students enrolled in MBBS Programme in the clinical phase participated. During the study period, students had surgery, family medicine, pediatrics, and medicine module. We invite the students personally at the end of each clinical module. Participation was entirely based on a voluntary basis; informed consent was obtained to fill the questionnaire. The data was collected from students using complete enumeration sampling method.

A self-structured questionnaire was used for data collection which was prepared by reviewing the literature and validated before final distribution. Psychometric properties and validity of the questionnaire were tested in two phases. In phase one, three specialists in the field, based on their comments, content validity and feedback modifications were evaluated. In phase 2, the reliability of the questionnaire was tested through Cronbach Alpha with the split-half method. The value of α was 0.71, which showed that the questionnaire had good reliability. The final questionnaire comprised of 21 items that were related to a range of topics directly relevant to feedback from students learning activities during modules. The questionnaire was divided into two main sections, demographic variables and three central themes, i.e., useful feedback, the purpose of feedback and the students believe. A 5-point Likert scale ranging from strongly disagree to agree strongly was used. In our college, we have continuous assessment during module and final assessment at the end of the module for promotion to next level; we use marks along with feedback for continuous assessment. Two open-ended questions were included in the questionnaire to have students’ opinion on current feedback process and their recommendation(s) for improving the feedback process.

The data was entered and analyzed using SPSS 23.0. Mean ± S.D was reported for quantitative variables. Frequencies and percentages were given for qualitative variables. Pearson Chi-Square and Fisher Exact Test were applied to observe an association between questions related to feedback, studying years and gender. Pearson Correlation was also applied to observe the relationship between GPA and mean scores of three central themes. Mean score was calculated by summing the ordinal scale variable/numbers of the item in each subgroup. Open-ended questions, however, were analyzed using thematic contents. Several themes were identified, and responses were categorized based on the consensus among two experts.

## RESULTS

A total of 145 students were studying in the clinical phase of which 121 students participated in the study with the response rate of 83.44%. Majority of the students were males (n=94; 77.7%) as compared to females (n=27; 22.3%). These students belong to 4^th^ (n=49; 40.5%), 5^th^ (n=47; 38.8%) and 6^th^ years (n=25; 20.7%) of their studies. The mean age of the students was 22.52 ± 1.19 years. The average GPA of all students was 3.61 ± 0.59. We perform an overall and then separate analysis for all three years for better understanding.

While exploring the student’s perception for useful feedback, students (n=55; 45.5%) disagreed with statements that feedback always contains marks. Half of the students (n=60; 49.6%) opined that they did not receive enough feedback. A significant association was observed between the year of study and response to these statements, the majority of the students studying in 4^th^ year agreed with these statements, whereas, most of the students studying in 5^th^ and 6^th^ year disagreed with them (p<0.05) respectively. While asking that whether feedback rarely provides them useful suggestions for improvement, the majority (n=75; 62.0%) of the students agreed, most of the students studying in the 6th year significantly agreed as compared to students studying in 4th and 5th year (p=0.026). Results are presented in Table-I.

**Table-I:** Students Perception of useful Feedback

	4^th^ year n (%)	5^th^ year n (%)	6^th^ year n (%)	Total	p-value
Feedback always contain marks/grades with it					
Disagree	13 (26.5%)	26 (55.3%)	16 (64%)	55 (45.5%)	0.009*
Neutral	7 (14.3%)	5 (10.6%)	3 (12%)	15 (12.4%)	
Agree	29 (59.2%)	16 (34%)	6 (24%)	51 (42.1%)	
Feedback is only useful when marks are low.					
Disagree	28 (57.1%)	28 (59.6%)	15 (60%)	71 (58.7%)	0.539
Neutral	11 (22.4%)	5 (10.6%)	3 (12%)	19 (15.7%)	
Agree	10 (20.4%)	14 (29.8%)	7 (28%)	31 (25.6%)	
In learning, Written feedback is more beneficial than the numbers only.					
Disagree	8 (16.3%)	16 (34.0%)	5 (20%)	29 (24.0%)	0.153
Neutral	14 (28.6%)	8 (17.0%)	3 (12%)	25 (20.7%)	
Agree	27 (55.1%)	23 (48.9%)	17 (68%)	67 (54.4%)	
Constructive criticism is needed for students to improve their learning.					
Disagree	4 (8.2%)	13 (27.7%)	6 (24%)	23 (19.0%)	0.068
Neutral	12 (24.5%)	5 (10.6%)	5 (20%)	22 (18.2%)	
Agree	33 (67.3%)	29 (61.7%)	14 (56%)	76 (62.8%)	
The trend of feedback should be more positive by the tutor(s).					
Disagree	16 (32.7%)	17 (32.6%)	13(52%)	46 (38.0%)	0.262
Neutral	0 (0.0%)	0 (0.0%)	0 (0.0%)	0 (0.0%)	
Agree	33 (67.3%)	30 (63.8%)	12(48%)	75 (62.0%)	
Tutor(s) provided me with enough written feedback.					
Disagree	16 (32.7%)	28 (59.6%)	16 (64.0%)	60 (49.6%)	0.047*
Neutral	3 (6.1%)	2 (4.3%)	1 (4.0%)	6 (5.0%)	
Agree	30 (61.2%)	17 (36.2%)	8 (32%)	55 (45.5%)	
Feedback rarely provides me with useful suggestions for improvement.					
Disagree	8 (16.3%)	12 (25.5%)	3 (12%)	23 (19.0%)	0.026*
Neutral	15 (30.6%)	7 (14.9%)	1 (4%)	23 (19.0%)	
Agree	26 (53.1%)	28 (59.6%)	21 (84%)	75 (62.0%)	

* statistically significant at 5% level of significance

When asked for the students understanding of the purpose of feedback, majority of students (n=76; 62.8%) agreed that feedback is a two-way process. Many students(n=58; 47.9%) agreed that feedback helped them to find out their expected performance, students studying in 4^th^ year disagreed more as compared to students studying in 5^th^ and 6^th^ year (p=0.021). Many students (n=63; 52.1%) think that tutor don’t provide opportunity to discuss their weaknesses, a significant association exists between the year of study and this response, 6^th^ year students agreed that they have a chance for active discussion while 4^th^ and 5^th^ year students disagree with them (p<0.05) respectively. A significant number of student (n=54; 44.6%) were of the opinion that the feedback is not helping them to identify areas for improvement. Results are presented in Table-II.

**Table-II:** Students perception of Purpose of Feedback.

	4^th^ year n (%)	5^th^ year n (%)	6^th^ year n (%)	Total n (%)	p-value
Feedback is a two-way process in which I am involved.					
Disagree	8 (16.3%)	8 (17%)	5 (20%)	21 (17.4%)	0.009*
Neutral	16 (32.7%)	8 (17%)	0 (0.0%)	24 (19.8%)	
Agree	25 (51%)	31 (66%)	20 (80%)	76 (62.8%)	
Feedback reflects the efforts I had put into the activities.					
Disagree	14 (28.6%)	16 (34%)	15 (60%)	45 (37.2%)	0.142
Neutral	11 (22.4%)	10 (21.3%)	3 (12%)	24 (19.8%)	
Agree	24 (49%)	21 (44.7%)	7 (28%)	52 (43.0%)	
Feedback allows me to have an active discussion with the tutors about my weakness					
Disagree	26 (53.1%)	30 (63.8%)	7 (28%)	63 (52.1%)	0.034*
Neutral	8 (16.3%)	5 (10.6%)	3 (12%)	16 (13.2%)	
Agree	15 (30.6%)	12 (25.5%)	15 (60%)	42 (34.7%)	
The Feedback helps me to identify areas for improvement.					
Disagree	24 (49%)	25 (53.2%)	5 (20%)	54 (44.6%)	0.013*
Neutral	10 (20.4%)	9 (19.1%)	3 (12%)	22 (18.2%)	
Agree	15 (30.6%)	13 (27.7%)	17 (68%)	45 (37.2%)	
Feedback helps me to engage in the process of learning and making an action plan.					
Disagree	8 (16.3%)	8 (17%)	7 (28%)	23 (19.0%)	0.714
Neutral	8 (16.3%)	9 (19.1%)	5 (20%)	22 (18.2%)	
Agree	33 (67.3%)	30 (63.8%)	13 (52%)	76 (62.8%)	
Feedback helps me to assess self-learning and reflects on my improvement.					
Disagree	7 (14.3 %)	9 (19.1%)	7 (28%)	23 (19.0%)	0.474
Neutral	9 (18.4 %)	10 (21.3%)	2 (8%)	21 (17.4%)	
Agree	33 (67.3 %)	28 (59.6%)	16 (64%)	77 (63.3%)	
Feedback helps me to differentiate between my performance and expected performance					
Disagree	21 (42.9%)	15 (31.9%)	3 (12%)	39 (32.2%)	0.021*
Neutral	12 (24.5%)	8 (17%)	4 (16%)	24 (19.8%)	
Agree	16 (32.7%)	24 (51.1%)	18 (72%)	58 (47.9%)	

* Statistically significant at 5% level of significance.

Moreover, we assess students believes regarding feedback. Students (n=55; 45.5% and n=56; 46.3%) disagreed that the written feedback has a relationship with the given marks and explain their performance and improvement area. The students studying in the 6^th^ year significantly disagreed more with these statements as compared to 4^th^ and 5^th^-year students (p<0.05) respectively. While asking about feedback according to published assessment criteria and whether the feedback is going to help them in scoring more marks in the final assessment, majority students (n=69; 57.0% and n=73; 60.3%) agreed. The students studying in the 4^th^ year significantly agreed as compared to students studying in 5^th^ and 6^th^ year (p<0.05) respectively. Most of the 6^th^ year students agreed that limited written feedback on learning activities is the reason for frustration as compared to 4^th^ and 5^th^ year students (p = 0.035). Results are presented in Table-III.

**Table-III:** Students believes about written Feedback

	4^th^ year n(%)	5^th^ year n(%)	6^th^ year n(%)	Total n (%)	p-value
Feedback I received has a relationship with the given marks					
Disagree	18 (36.7%)	23 (48.9%)	14(56.0%)	55 (45.5%)	0.027*
Neutral	11 (22.4%)	16 (34.0%)	2 (8.0%)	29 (24.0%)	
Agree	20 (40.8%)	8 (17.0%)	9 (36.0%)	37 (30.6%)	
Feedback adequately explain why a mark was given and what would be required for improvement					
Disagree	12(24.5%)	26 (55.3%)	18(72.0%)	56 (46.3%)	0.001*
Neutral	9 (18.4%)	4 (8.5%)	0 (0.0%)	13 (10.7%)	
Agree	28 (57.1%)	17 (36.2%)	7 (28.0%)	52 (43.0%)	
Limited feedback on given marks is the reason for frustration					
Disagree	12 (24.5%)	3 (6.4%)	1 (4%)	16 (13.2%)	0.035*
Neutral	10 (20.4%)	10 (21.3%)	3 (12%)	23 (19.2%)	
Agree	27 (55.1%)	34 (72.3%)	21 (84%)	82 (67.8%)	
Feedback is used to justify or explain the given marks					
Disagree	9 (18.4%)	14 (29.8%)	6 (24%)	29 (24.0%)	0.332
Neutral	14 (28.6%)	7 (14.9%)	3 (12%)	24 (19.8%)	
Agree	26 (53.1%)	26 (55.3%)	16 (64%)	68 (56.2%)	
In feedback, it did not appear whether the marks were high or low.					
Disagree	11 (22.4%)	22(46.8%)	5 (20%)	38 (31.4%)	0.073
Neutral	12 (24.5%)	7 (14.9%)	5 (20%)	24 (19.8%)	
Agree	26 (53.1%)	18 (38.3%)	15 (60%)	59 (48.8%)	
Feedback is according to assessment criteria as mentioned in the module.					
Disagree	4 (8.2%)	13 (27.7%)	10 (40%)	27 (22.3%)	0.010*
Neutral	12 (24.5%)	11 (23.4%)	2 (8%)	25 (20.3%)	
Agree	33 (67.3%)	23 (48.9%)	13 (52%)	69 (57.0%)	
Feedback is going to help me in scoring more marks in the final assessment.					
Disagree	6 (12.2%)	9 (19.1%)	16 (64%)	31 (25.6%)	<0.001*
Neutral	6 (12.2%)	9 (19.1%)	2 (8%)	17 (14.0%)	
Agree	37 (75.5%)	29 (61.7%)	7(28%)	73 (60.3%)	

* statistically significant at 5% level of significance.

**Fig.1:**
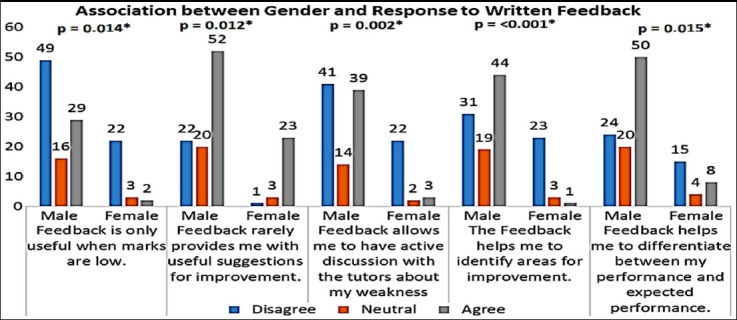
Association between Gender and responses.

Female students significantly disagreed more as compared to the male students regarding statements presented in Fig.1 with p<0.05. Rest of the questions had no significant association with gender (p>0.05).

**Fig.2:**
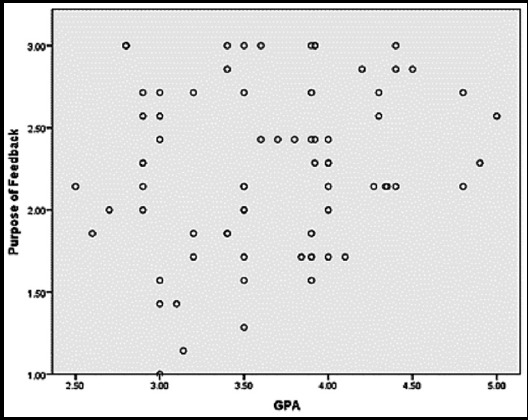
Scatter Plot of GPA and Mean Score of Purpose of Feedback.

A positive correlation was observed between GPA and mean score of the purpose of feedback (r=0.270, p=0.020). However, the mean score of student’s perception for useful feedback and the student’s belief for written feedback was not significantly correlated with GPA (p > 0.05) respectively.

Two open-ended questions were included to have a qualitative opinion of students on current feedback process and suggestions for improvement. Students shared their opinion on current feedback process and several suggestions for improvement were made. Results are presented in Table-IV.

**Table-IV:** Students Opinion and Suggestions for Current Feedback Process.

Opinion	Number of comments
Appropriate feedback is not received during activities	12
The marks were given without qualitative written comments	31
We received the feedback in the form of oral comments	6
Feedback is usually given to those with low marks	5
Feedback is usually received in the form of compliments or critique, without any details	3
Strengths also need to be highlighted	5
Tutors do not provide feedback on weakness and how to improve them	11
The feedback received varies between tutor-to-tutor; lacking structured pattern	9
*Suggestions*	
There should be a uniformly structured feedback system by faculty	13
Written comments are better than oral feedback	6
Must highlight weakness and area for improvement	23
There is a need to receive Comments from peers and year coordinator	3
There should be an electronic feedback system	2
Student need session for interpretation and how to utilize comments	16

## DISCUSSION

Students develop a perception for feedback by a complex mechanism, including learning environment, educator role and self-belives to perceive, interpret and use the feedback.^12^ Better learning is achieved by constructive feedback. Our study report that 45.5% of the students did not link feedback with marks, possibly our assessment method is the reason for this perception, there is a gradual shift towards formative assessment from junior class to senior class where we provide corrective feedback in activities like mini-cex,case-based discussion, etc. This is reflected as statistically significant maturity difference in responses(p=0.009), senior class perceived written feedback more beneficial.This finding is similar to another study where senior students appreciate feedback more and self-directed learner by utilizing the feedback.^13^

Interestingly we find that majority 58.7% students from all three cohorts consider written feedback very useful for their learning, and they don’t link receiving feedback only with low marks. The finding suggests that students give importance to comments and in search of feedback to understand their mistakes and obtain diagnostic information.

Insufficient feedback either in term of quality or frequency is reported globally as well as in Saudi arabaia.^9^,^14^ Almost half 49.6% of our students responded that they do not receive enough feedback. We also noticed the highest response to open-ended comments regarding insufficient feedback; students suggested to include their weakness as well as strengths in written comments. Faculty responded that they value and provide sufficient feedback regularly but student’s response to feedback is unsatisfactory.^7^,^14^ Also most 62% of student reported that feedback rarely provides them with useful suggestions for improvement.Students suggested in the open-ended response to provide written comments rather than informal oral comments on their performance. The absence of a clear system of feedback; inadequate skills for delivering effective feedback and language barriers are reasons for students dissatisfaction.^15^ The finding creates a need of having a critical analysis of the feedback process in clinical years by introducing a structured feedback strategy with emphasis on an improvement plan for learning.

Feedback is not only for students but can also help the tutors to regulate and upgrade the learning process.^16^ Our study revealed that students understand the purpose of feedback as majority 62.8% students agrees for two-way nature of feedback. More than half 52.1% of the students portrayed that feedback does not allow them active discussion with tutors leading to one-way learning. This can be interpreted as feedback-seeking behavior. Students who sought feedback frequently can improve their work performance by setting feedback-based goals.^17^

When asked whether current feedback help them to identify the area for improvement, students from all three cohorts responded that one-way feedback does not help them to improve (p=0.013), but they agree that they can differentiate their expected performance from feedback (p=0.021). students in the open-ended responses emphasized that written feedback needs corrective measures from feedback providers, as the process should not only focus on highlighting the weakness and strengths but it should provide a roadmap for progress to upgrade oneself towards learning goals.^18^

Summative assessment delivered to the students with limited written comments and highlighting forward-looking guidance can cause anxiety and demoralization leading to poor future performances.^19^ Nearly half the students disagree that feedback has a relationship with marks they received during the assessment (p=0.027), neither educator provides them reasons for low marks nor comments for improvement. A large number 67.8% of student reported frustration because of limited feedback. Educators should explain their feedback approach explicitly to students for grading their transcripts.^20^ For instance, the majority of the students in our study agreed that criteria were clear in the module guide and feedback helped them to score high marks in the final assessment. This may be attributed to the difference in provision of feedback and students believes on the usefulness of feedback (p<0.001). Also, students highlight that there is a variation of feedback between tutor to tutor and there is a demand to have some structured process for feedback

The study has provided us insight into the students believes and perception for feedback as they find feedback helpful in their learning in different clinical modules. In open-ended response, they suggested for activities to understand and utilize the feedback effectively. There is a need for explaining the marking scheme for future improvement and keep a follow-up that students understand and utilize these marks and written comments for their learning growth.

### Limitations of the study

Our medical college is a newly established institute with a limited number of students. Although the data were collected during different clinical modules, still the study had a small sample size. We do not have baseline study on the student’s perception of feedback for comparison as well as limited local literature available in the region.

## CONCLUSION

Majority of students in this study were aware of the purpose of feedback. There was also a noticeable maturity difference from junior batch towards senior batch regarding giving value to written feedback and considering it a tool for enhancing the learning process. Students with higher GPA were apparently more aware of the purpose of feedback. The results highlight the need for more frequent and structured feedback mechanism, there is also a need for faculty engagement in training for understanding and delivering the feedback to the students.
